# Supplementary material for: Understanding political communication and political communicators on Twitch
Source: PLoS One. 2024 Nov 27;19(11):e0314429. doi: 10.1371/journal.pone.0314429 (PMC11602016; doi:10.1371/journal.pone.0314429)
Supplement: S1 Appendix — (PDF) [file pone.0314429.s001.pdf]

Appendix for "Understanding Political Communication and Political Communicators on Twitch"

Section A. Game name identification The first step of the process is getting to know what "game-names" political streamers use. "Game-names" are tags of Twitch live-streaming that refer to what streamers are broadcasting. As Twitch is a mainly game-oriented platform, most game-names are the names of the games they are playing, such as League of Legends and Fortnite. However, there are game names that are not directly related to gaming activities, ranging from "Just Chatting" to "Cooking."

It is quite apparent that streamers who stream with the game name "Politics" can be classified as political streamers and can be added to the list. Based on the information from the site "Twitchmetrics," which provides various information on Twitch streamers including rankings by category based on the information received through the Twitch API, I learned that political streamers also use the game name "Talk Shows and Podcasts" [1]. However, some streamers stream political content without using "Politics" or "Talk Shows and Podcasts" as the game name of their streams.

A notable example is Hasanabi, who never broadcasts with "Politics" but instead uses "Just Chatting" as the game name when discussing political issues. This raises the possibility of finding political streamers among those using the "Just Chatting" tag. However, many streamers who do not explicitly stream gaming activities—some even stream their gaming activity under "Just Chatting"—choose this game name for a variety of content, from talk shows to vlogging their travels. Consequently, retrieving data on streamers who use the "Just Chatting" game name through the Twitch API would yield a diverse list of topics.

Before including the "Just Chatting" game name in the list of potential political streamers, I conducted a preliminary analysis of streamers using this tag. Using the Twitch API's "Get Streams" function, I retrieved a list of streamers who were streaming in English under the game name "Just Chatting" at a specific time. I identified 873 streamers during this process, stopping when the list ended with a streamer with a single viewer count. The "Get Stream" request retrieves various information about ongoing streams, including the user ID of the streamer, viewer count, the title of the stream, and the URL of a thumbnail image.

To determine whether the streams cover political content, I found that thumbnail images and stream titles can be helpful indicators. By using the URL information from the retrieved data, I downloaded all thumbnails of the 873 streams, which serve as the visual representation of what viewers were watching at the time of the request. By combining visual information with text information from the streaming titles, I was able to identify some political streams.

In reviewing the titles of these streams, several included political terminology. For example, one title discussed the "DNC working to block Nina Turner," while another asked, "Where are our freedoms?." The titles of streams included terms such as "Mayor's Race", "freedom", "LGBTQ+", and "Candace Owens vs. Kim Klacik." Overall, the analysis of both the textual information from titles and the visual elements suggested that there are indeed political streamers broadcasting under the game name "Just Chatting."

Section B. Coding rule for ideology and gender from profile information of political streamers The streamers' ideology and gender were inferred based on the following coding rules:

1. Ideology:
   (a) If a streamer explicitly self-declares their ideology (e.g., "I am a leftist"), I coded their ideology accordingly.
   (b) If a streamer makes a very explicit political statement or supports a politician with a clear ideology (e.g., Marxism, Libertarianism), I coded their ideology based on that statement.
   (c) If a streamer supports a clearly partisan group (e.g., NRA), I coded their ideology based on the affiliation.
2. Gender:
   (a) If a streamer self-declares their gender using pronouns (e.g., "she/her"), I coded their gender accordingly.
   (b) If a streamer uses an explicitly gender-revealing name (e.g., Claire, Jonathan), I coded their gender based on the name.
   (c) If a streamer uses explicit words related to gender (e.g., mom, dad), I coded their gender based on these terms.

Table C1. ML Models Performance

Model	Precision	Recall	F-1
Logit + Count	0.866	0.718	0.765
Logit + TFIDF	0.953	0.554	0.587
XGBoost + Count (learning rate = 0.5)	0.839	0.740	0.778
XGBoost + TFIDF (learning rate = 0.5)	0.851	0.735	0.776

Section C. Supervised machine learning classifier for identifying political streamers I have trained logistic regression and XGBoost classifiers using the train data. And I have specified models with the count and TF-IDF vectorizer for each algorithm. As the target value of the dataset is infrequent, I have used precision, recall, and F-1 score, which are known to be more appropriate to evaluate imbalanced data, to evaluate the performance of classifiers. Table A1 shows the performance of the 5-fold cross-validation results of each model. XGboost models mostly outperform logistic regression models in all three criteria, while it is hard to say which vectorizer is outperforming. I have used the model with TFIDF vectorizer for machine labeling the rest of the unlabeled data as it is well known to perform better to deal with complex text data [2]. Through this process, I was able to identify additional 48 political accounts from 45,477 just chatting streamer accounts. Adding 550 political accounts identified during the hand-coding process, I was able to identify 598 political accounts in total. After addressing duplicates due to the flexibility in game names during streaming, I was able to identify a total of 574 unique political streamers on Twitch. This process involved removing streamers who appeared in multiple categories, such as those who streamed under both “Just Chatting” and “Politics” game names.

Section D. Coding rule for political topic classification

1. **Identifying political topics:**

    - I identified a topic as political if one or more predefined politically relevant terms were among the top 15 keywords within a topic.
    - **Predefined categories of politically relevant terms:** These categories encompass various types of terms commonly associated with political discourse, ensuring a comprehensive identification of political topics:
        - **General political terms:** Includes terms related to governance, ideologies, social issues/conflict, politicized topics, and political concepts (e.g., "democracy," "vote," "communism," "capitalism", "drug", "gender", "feminism").
        - **Names of political figures:** Includes notable individuals associated with politics (e.g., "Trump," "Biden," "AOC").
        - **State or country names:** Refers to geographical entities relevant to political discussions (e.g., "USA," "Russia," "Ukraine").
        - **Political movements or events:** Encompasses terms related to specific movements or significant events (e.g., "BLM," "protest," "terrorism").
        - **International issues:** Terms related to global issues affecting politics and conflicts or political situations between nations (e.g., "climate," "trade," "Ukraine war," "NATO").

2. **Topic naming and boundaries:**

    - Each identified political topic was manually reviewed, and a descriptive label was assigned based on the most prominent political keywords.
    - **Example**: A topic containing "trump," "woman," and "gun" was named *Trump and gender*, while a topic with "sex," "gender," "drug", and "legal" was named *Gender and drug.*

3. **Topic aggregation into categories:**

    - After the topics were named, they were aggregated into seven broader categories based on thematic similarities:
        (a) International issues
        (b) US politics
        (c) Identity politics
        (d) Ideological debate
        (e) Public health and politics
        (f) Environmental issues
        (g) Politics in general
    - **Guidelines for categorization**: To reduce ambiguity between similar categories (e.g., Identity Politics vs. US Politics), I followed specific guidelines:
        - **US Politics**: Topics were categorized under US Politics if they centered on American political institutions, parties, or figures (e.g., keywords like "Trump," "Democrat," "Republican").

  - **Identity Politics**: Topics were categorized as Identity Politics if they focused on political issues related to race, gender, or identity groups (e.g., keywords like "gender," "ethnicity"). However, if there was a possible overlap between Identity Politics and US Politics, the topics were conservatively categorized under US Politics.
  - **International Issues**: Topics mentioning non-US political events, conflicts, or figures (e.g., "Putin," "Ukraine," "Turkey") were categorized under International Issues.
  - **Ideological Debate**: Topics that did not fall under US Politics, Identity Politics, or International Issues, but involved discussions around political ideologies, beliefs, or philosophical arguments (e.g., "socialism," "libertarianism," "conservatism").
  - **Public Health and Politics**: Topics that did not fall under US Politics, Identity Politics, or International Issues, but involved political discussions surrounding health-related issues, including healthcare policies, pandemics, or public health movements (e.g., "COVID-19," "healthcare," "vaccination").
  - **Environmental Issues**: Topics that did not fall under US Politics, Identity Politics, or International Issues, but focused on environmental concerns and policies affecting political discourse (e.g., "climate change," "pollution," "sustainability").
  - **Politics in General**: Topics that did not fall under none of the six topics but addressed broader political themes or generic political discussions (e.g., "political news," "elections," "civic engagement").
- **Example**: A topic containing "trump," "woman," and "gun" was categorized under *US politics*, while a topic with "sex," "gender," "drug", and "legal" was categorized under *Identity politics*.

Section E. Extensive lists of political topics

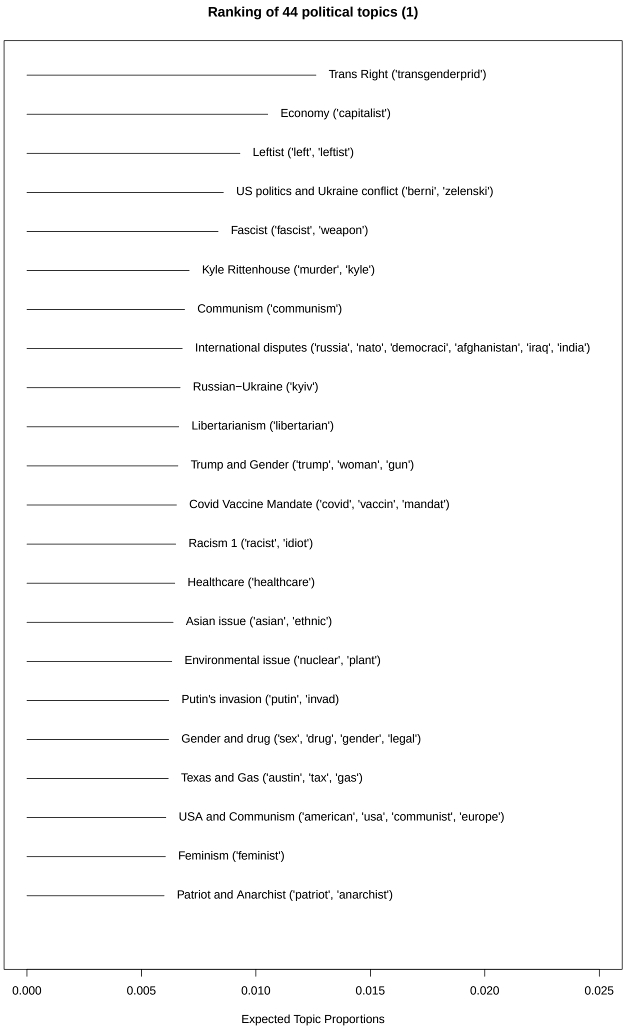

Fig E1. Identified Political Topics (1)

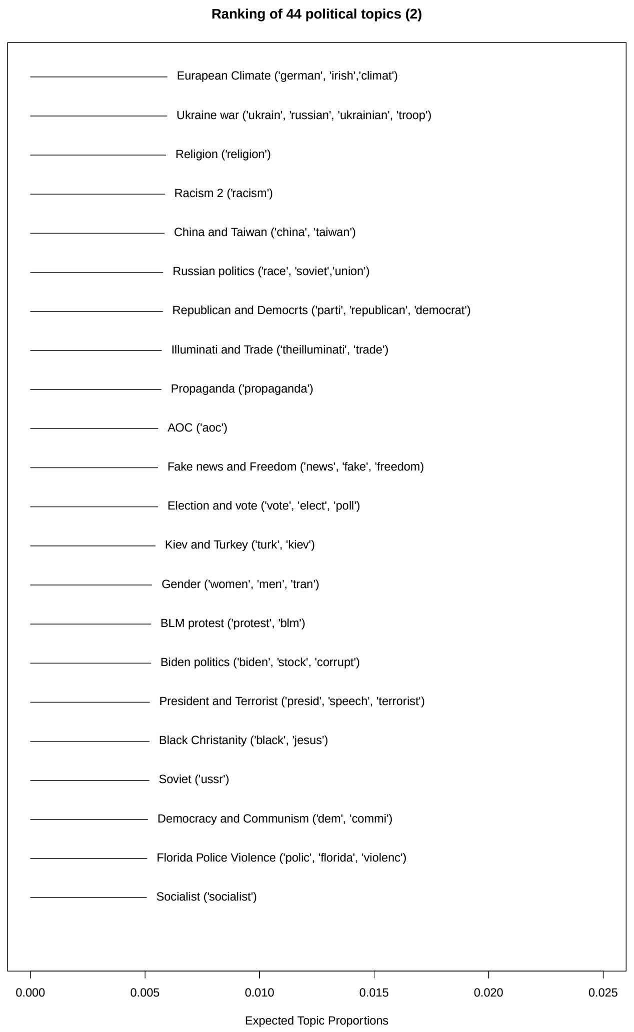

Fig E2. Identified Political Topics (2)

Section F. Full plots for 150 topics

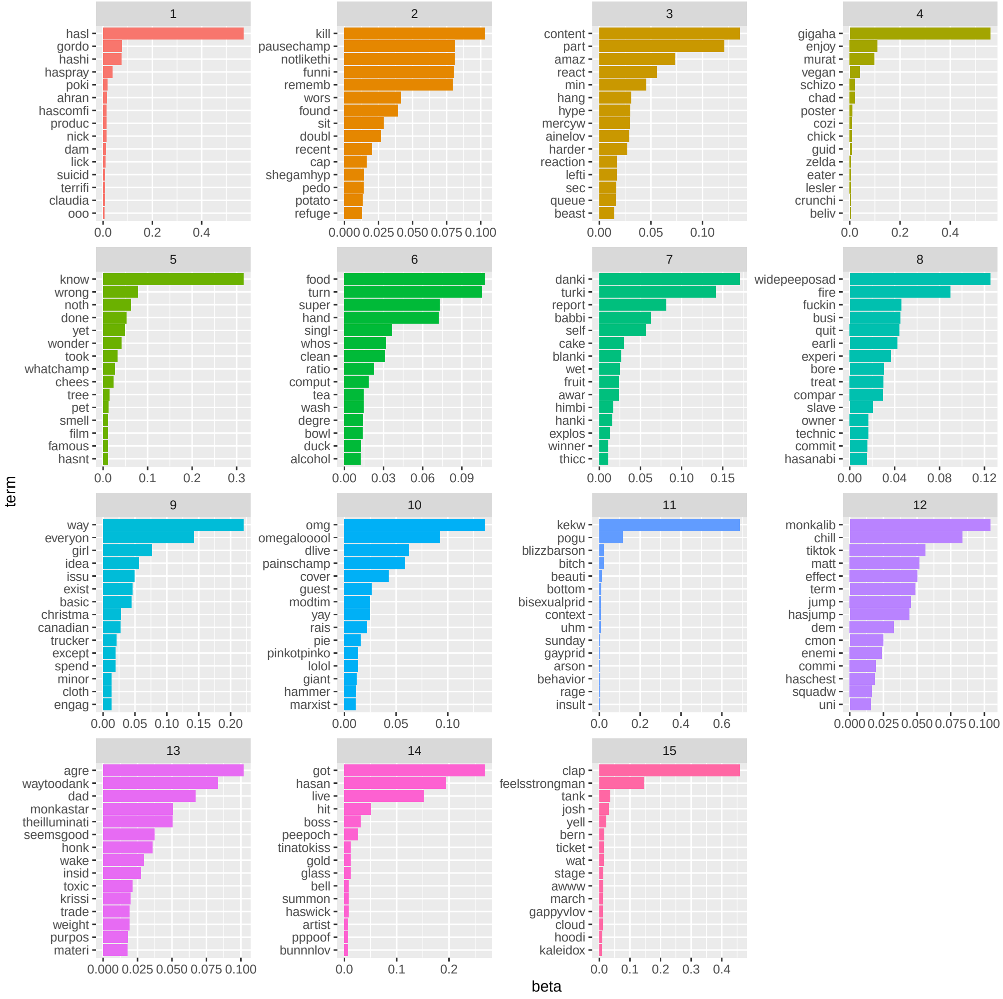

Fig F1. Total 150 topics (1)

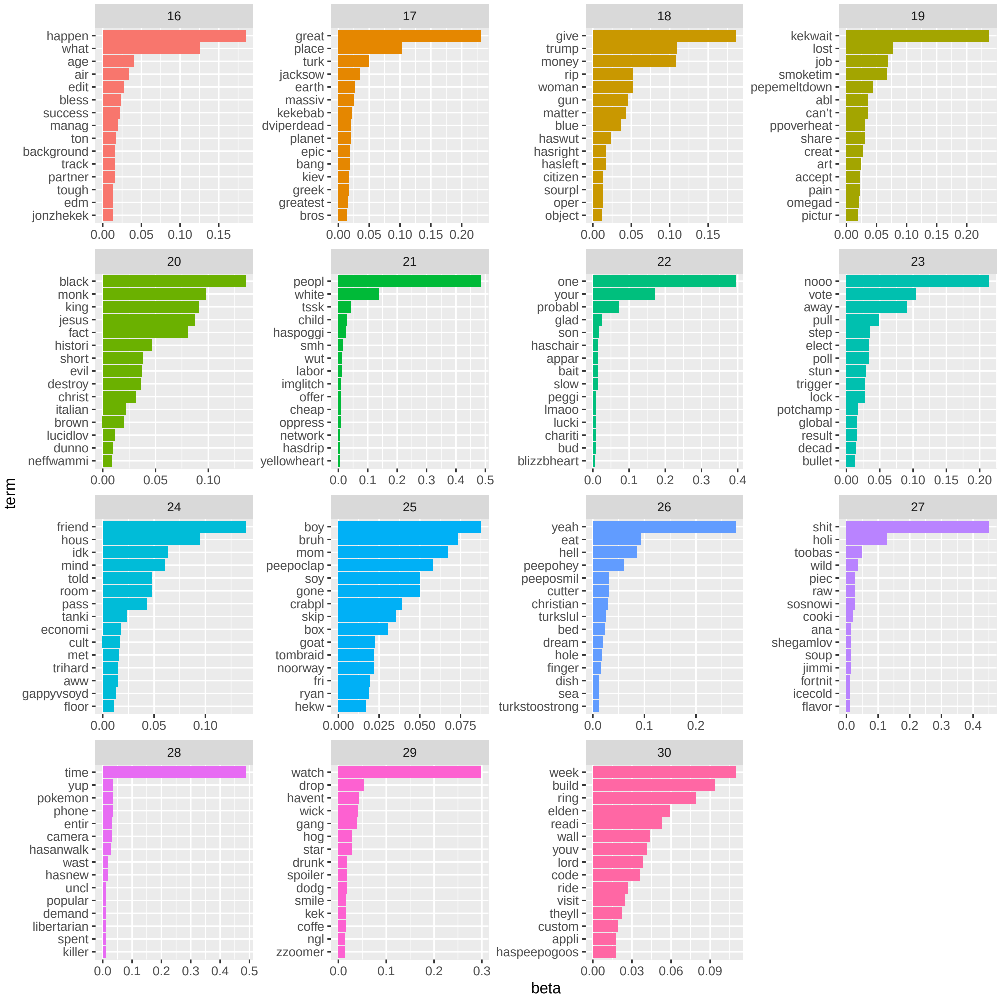

Fig F2. Total 150 topics (2)

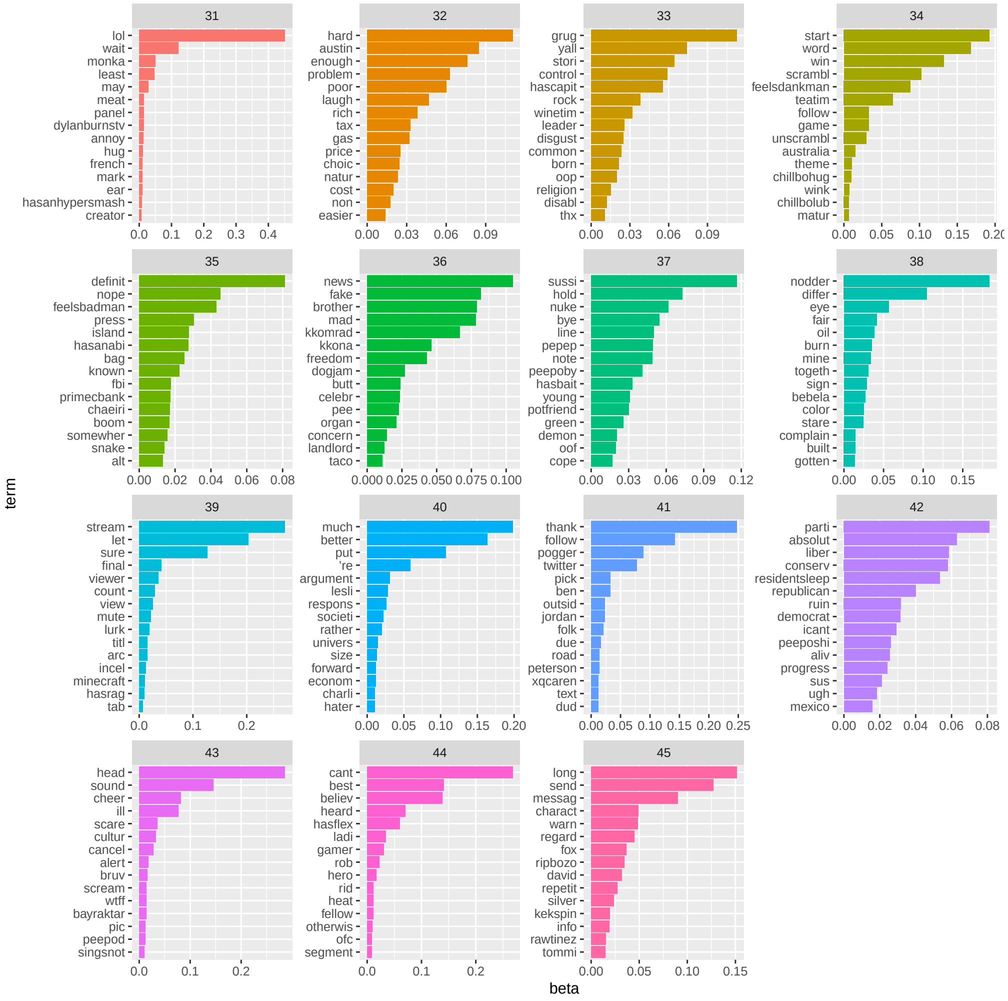

Fig F3. Total 150 topics (3)

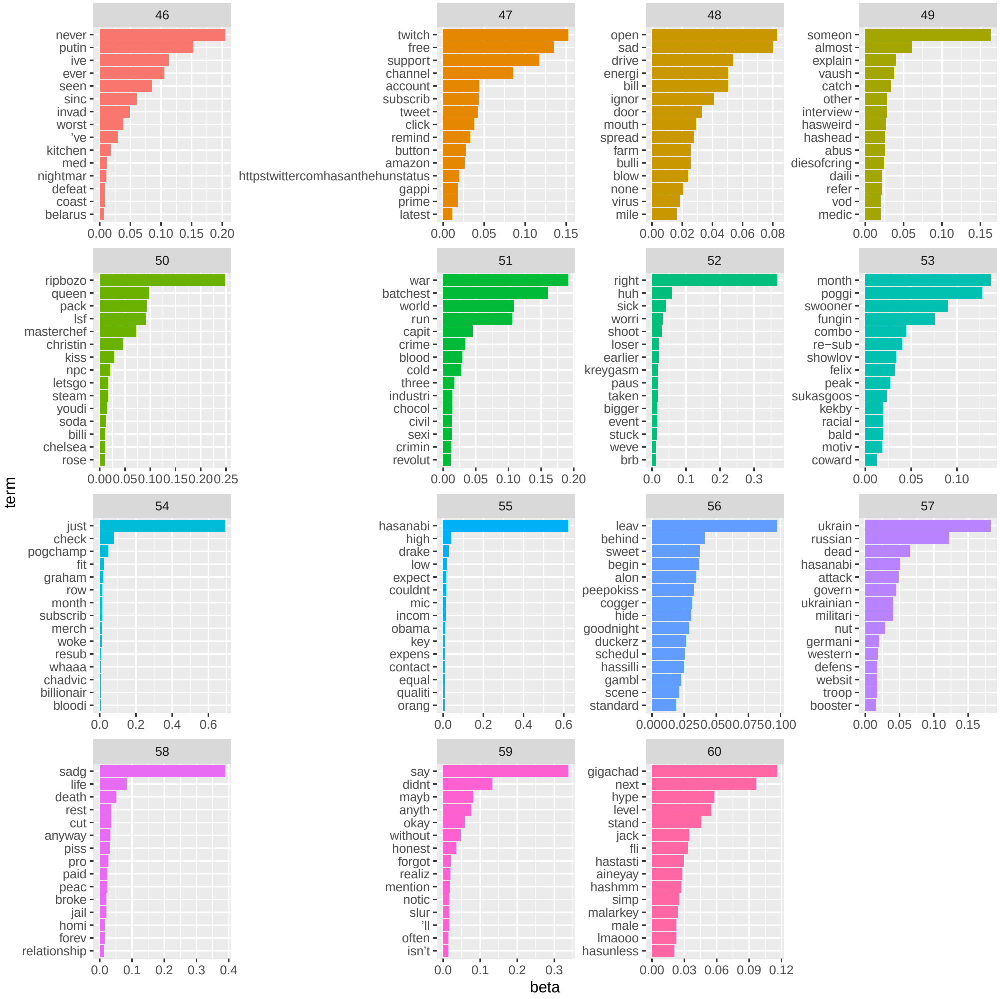

Fig F4. Total 150 topics (4)

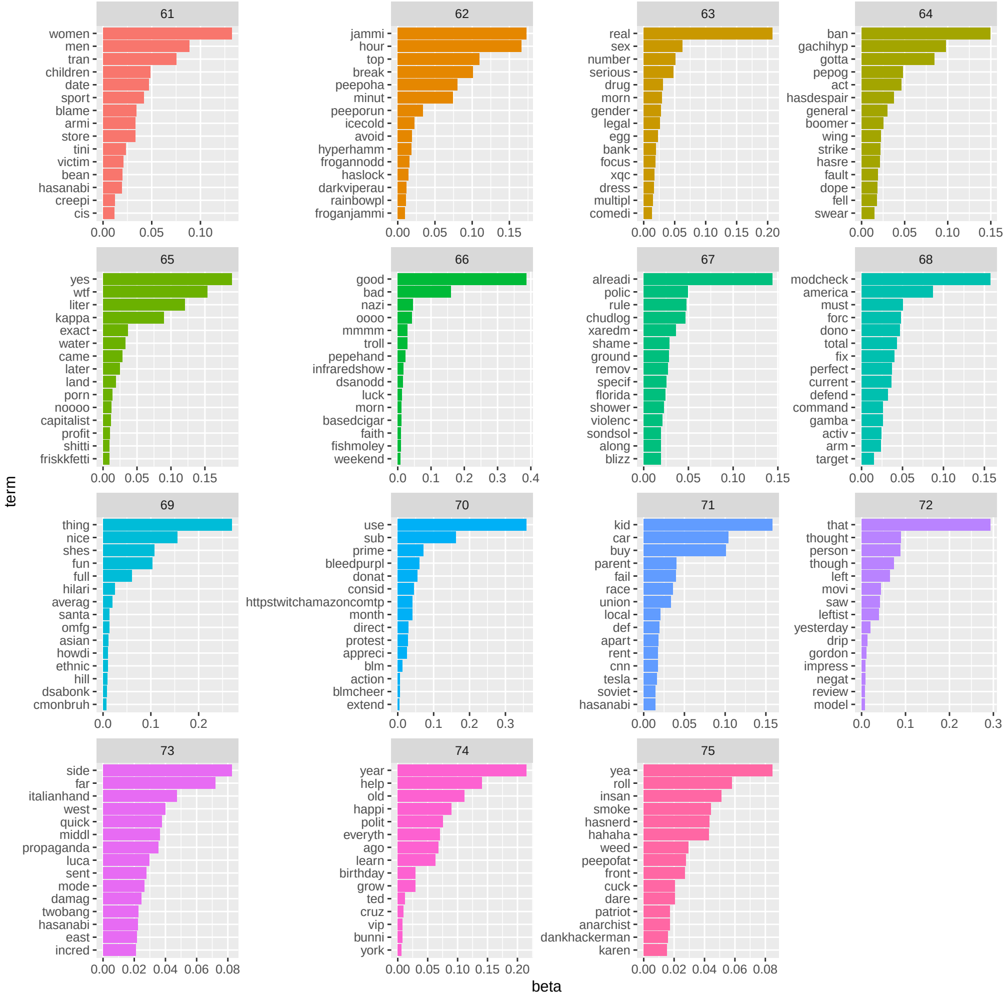

Fig F5. Total 150 topics (5)

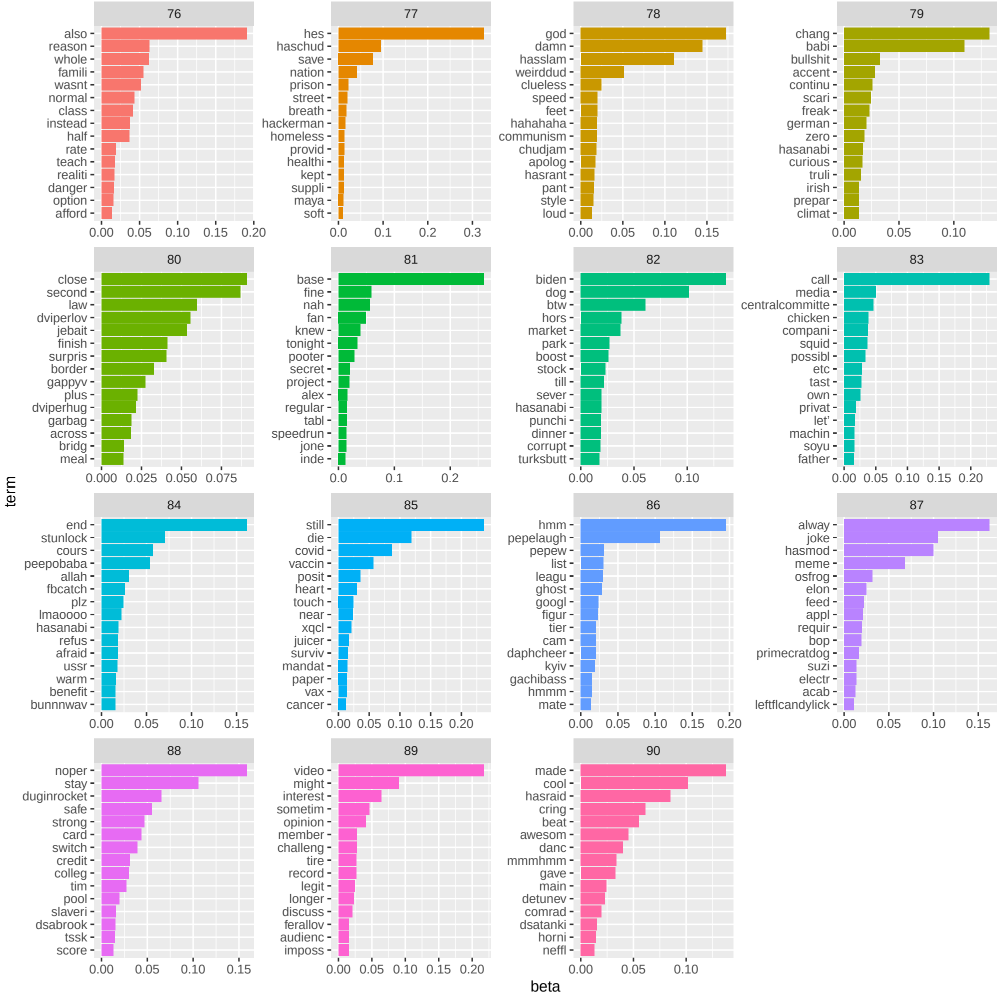

Fig F6. Total 150 topics (6)

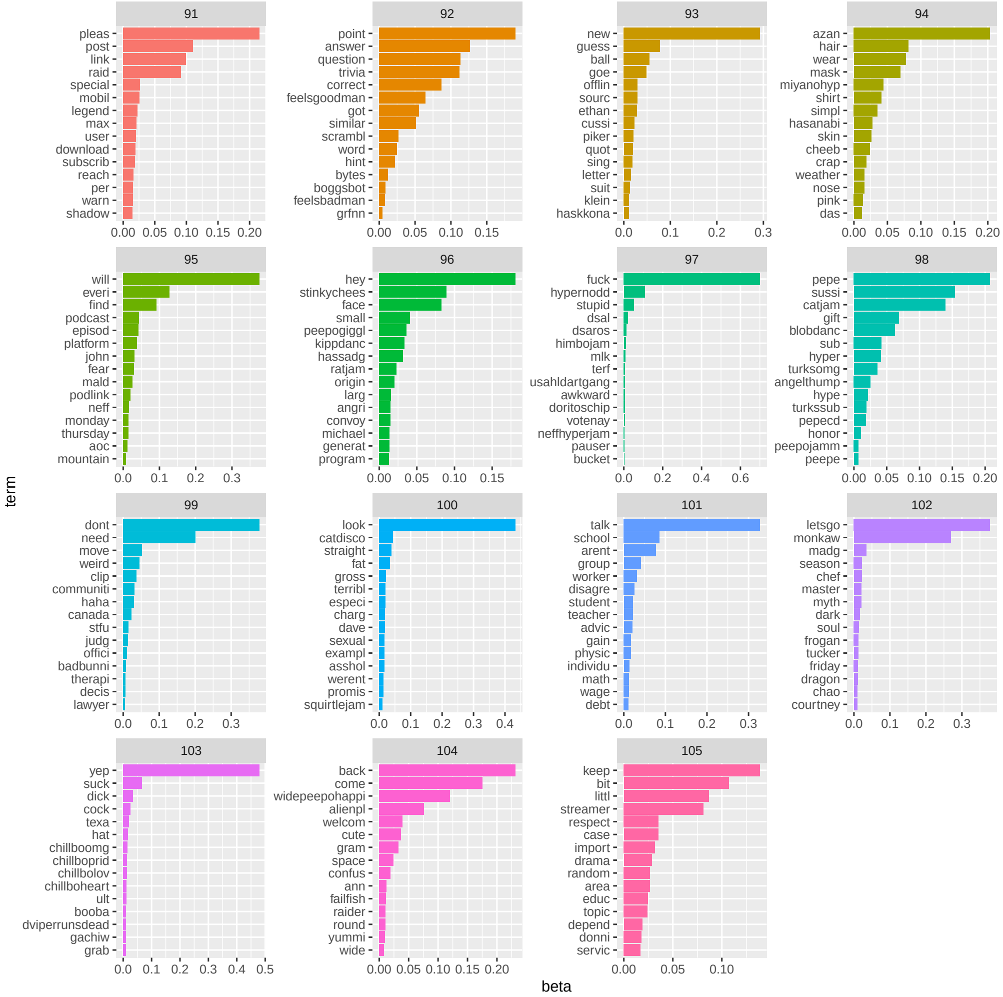

Fig F7. Total 150 topics (7)

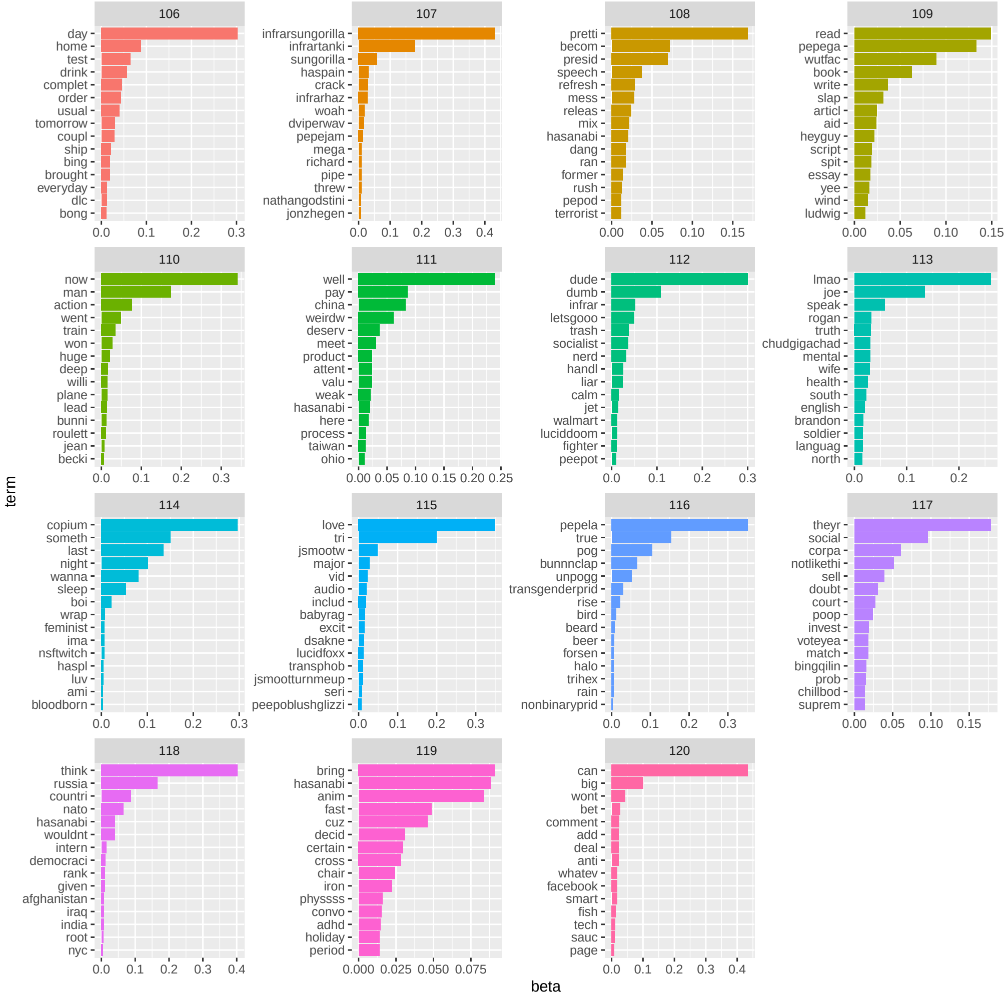

Fig F8. Total 150 topics (8)

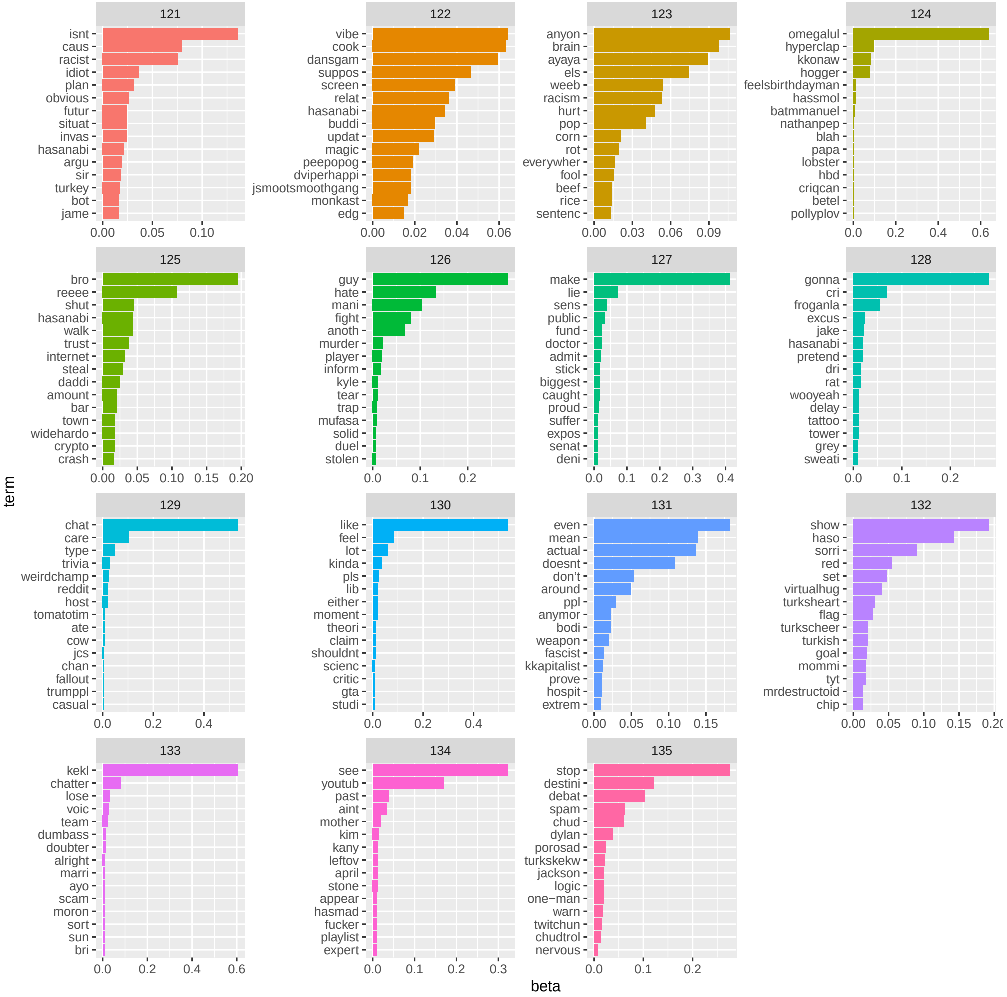

Fig F9. Total 150 topics (9)

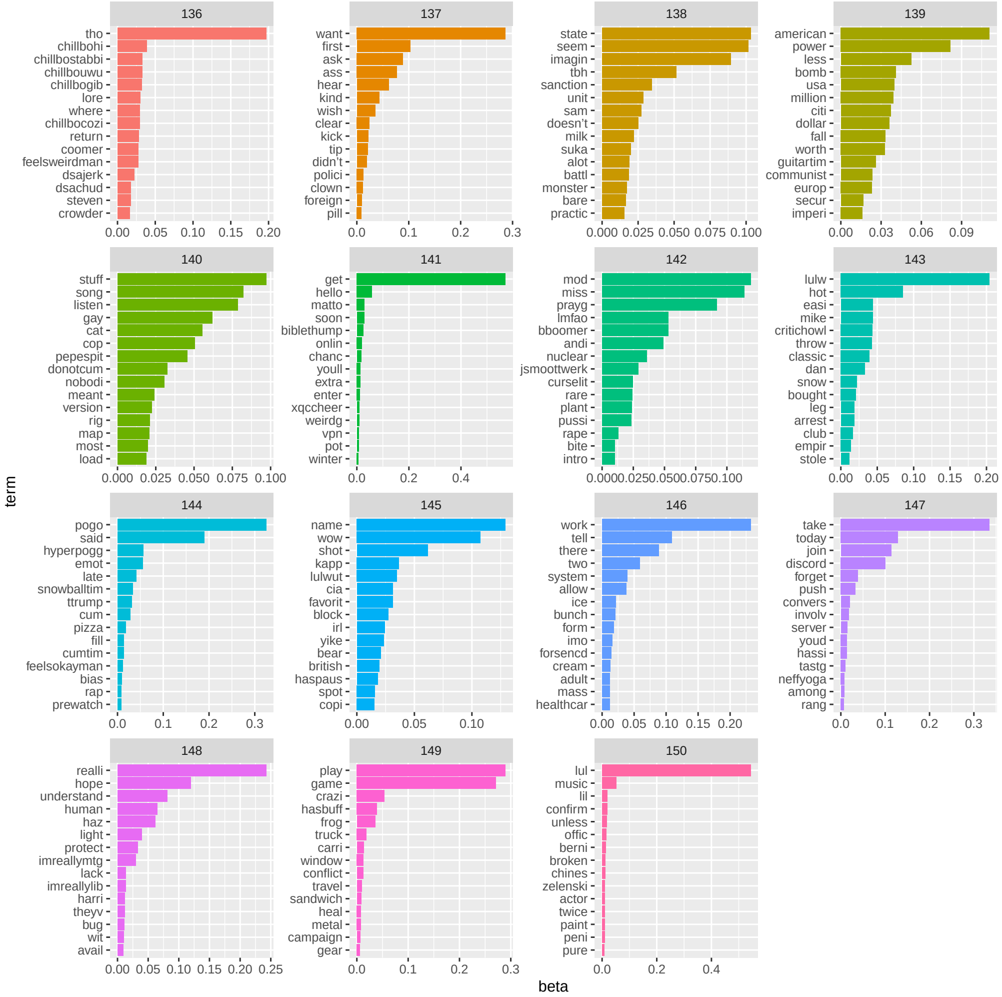

Fig F10. Total 150 topics (10)

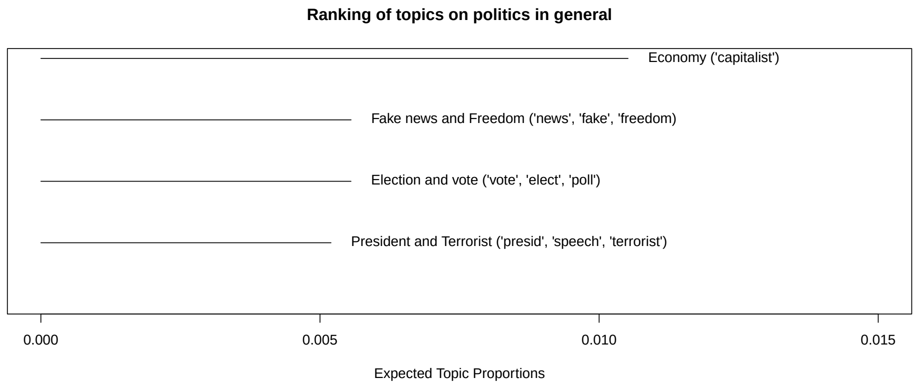

Fig G1. Topic Ranking: Politics in general

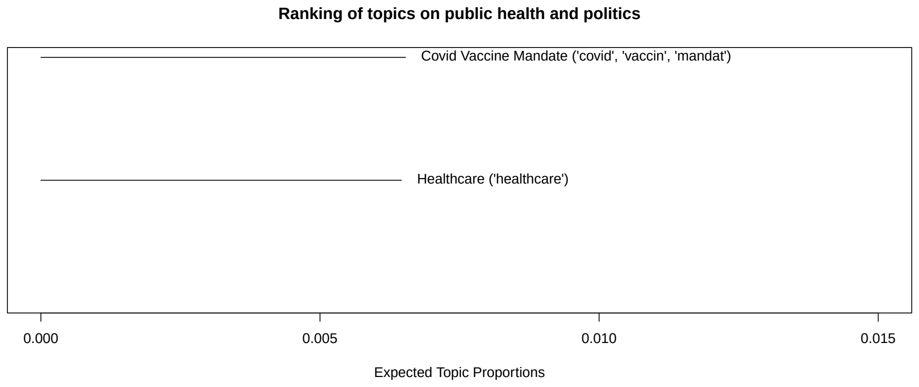

Fig G2. Topic Ranking: Public health and politics

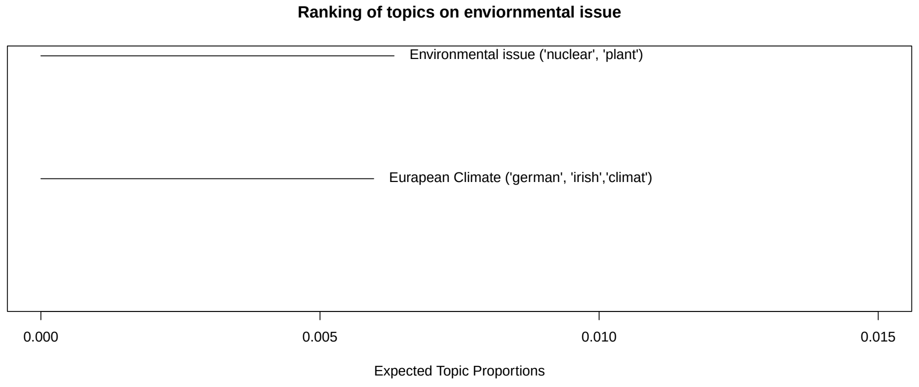

Fig G3. Topic Ranking: Environmental issue

Section G. Topic ranking for "Politics in general", "Public health and politics", "Environmental issue"

References

[1] Twitchmetrics. Twitchmetrics - Streamers and Games. twitchmetricsnet. 2022;.

[2] Ahmed T, Mukta SF, Al Mahmud T, Al Hasan S, Hussain MG. Bangla text emotion classification using LR, MNB and MLP with TF-IDF & CountVectorizer. In: 2022 26th International Computer Science and Engineering Conference (ICSEC). IEEE; 2022. p. 275–280.
