# Supplementary material for: Understanding political communication and political communicators on Twitch
Source: PLoS One. 2024 Nov 27;19(11):e0314429. doi: 10.1371/journal.pone.0314429 (PMC11602016; doi:10.1371/journal.pone.0314429)
Supplement: Attachment — Submitted filename: Response to Reviewers.pdf [file pone.0314429.s002.pdf]

Response to Reviewers

Dear academic editor and anonymous reviewers,

I want to express my sincere gratitude for valuable feedback and giving me a chance to revise my manuscript. The manuscript has been revised to address questions and concerns raised by the reviewers. The summary of the revisions made would be addressed in this letter with some detailed answers for the questions and suggestions from the reviewers. The comments are expressed in **bold** while the response is expressed in *italic*.The responses to each comment are as follows:

1. Editor-level/Journal requirement related issues:

From editor:
"As also mentioned by the reviewers, all the conclusions drawn in the paper should be clearly supported by the data. Additionally, the paper should facilitate the reproducibility of the results by providing easy access to the data that has been used in the context of this study."

Response: Thanks for the valuable comment. I have revised the manuscript in the way that the findings are properly supported by the data and acknowledge the limitations of the paper properly. Also, all the data related to the paper is now shared in Harvard Dataverse: https://doi.org/10.7910/DVN/AE86N1. I will also note this in the submission portal as well.

From Journal requirements:
"Please note that PLOS ONE has specific guidelines on code sharing for submissions in which author-generated code underpins the findings in the manuscript. In these cases, all author-generated code must be made available without restrictions upon publication of the work."

Response: All my codes for the paper is now up in Harvard Dataverse: https://doi.org/10.7910/DVN/AE86N1

"In your Methods section, please include additional information about your dataset and ensure that you have included a statement specifying whether the collection and analysis method complied with the terms and conditions for the source of the data."

Response: I added more description of the data and the data collection process complied with terms of conditions of Twitch API in the section 4. It can be found in line 310-315 in track-change version and line 242-247 in plain text version.

"We note that Figures 1,12 and A1 in your submission contain copyrighted images. All PLOS content is published under the Creative Commons Attribution License (CC BY 4.0), which means that the manuscript, images, and Supporting Information files will be freely

available online, and any third party is permitted to access, download, copy, distribute, and use these materials in any way, even commercially, with proper attribution.”

Response: All the copyrighted material is eliminated from the paper.

2. Comments from the reviewers:

Reviewer 1:

“The articles investigates the use of Twitch for political communication. The author provides a very detailed account of the methodology used (data collection via Twitch APIs, supervised classification, topic modeling, network analysis), through which he identifies a large set of political streams, classifies the recurring topics and analyses the interactions between the users. While very interesting quantitative results are provided, the discussion of these results could sometimes be deepened and more connected with the (extensive) literature review of the first sections. While some more methodological details are given in the Supporting information section, no link is provided to the raw data used in the study.”

Response: I want to start with saying thank you to the Reviewer for kind words with very userful comments. All the changes I made based on the specific recommendations from Reviewer 1 would be described in detail below. For this comment, the biggest concern I see is related to data sharing. As I mentioned above, all the raw data to reproduce the figures and results in paper is now up in the Harvard Dataverse (https://doi.org/10.7910/DVN/AE86N1). Also, I provided more extensive results that were not included in the previous version of the paper in the Supporting information section.

“Page 5, "live streaming on Twitch is even more efficient because the time spent for streaming is the same as the length of the show" -> the formulation is not very clear ; it also implies that streamers are not spending time preparing their performance before the stream.”

Response: Thanks for pointing this out. The reviewer’s point is absolutely right – I revised in the way that resolved the ambiguity the reviewer pointed out. It appears in the line 240-243 in track change version, line 176-179 in plain text version.

“Page 5, "Although not all streamers can earn a stable income -> It would be more fair to say that only a tiny minority of streamers can earn a stable income (data is hard to find, but it cannot exceed 1% of the streamers).”

Response: Thanks for pointing this out. I edited in a way that eliminates the possibility of misunderstanding. It appears in line 254 in track change version, line 188 in plain text version.

“Page 8, to properly answer the first question (Who are the political Twitch streamers ?), it would have been interesting to provide some insights on the identified profiles (based on the data actually collected through the API : registration date ? number of followers ? location ? age ? gender ?)”

Response: I want to thank the Reviewer for suggesting a very interesting and meaningful way to advance the article. I conducted the suggested analyses and added a new section “Descriptive features of political streamers.” The number of followers and location information was not able to be retrieved at all, and the age information was extremely limited (only 11 reported in their profile) to be analyzed. Although it is a bit limited as reported in the paper, I conducted descriptive analyses on registration date, view counts for profile, gender, and ideology of identified political streamers. It appears in line 391-423 in track change version, line 309-3 41 in plain text version.

“Some figure captions are a bit short (ex : fig 2, fig 6, fig 11) and could be expended to guide the interpretation of the diagrams.”

Response: I deeply appreciate the reviewer for pointing this out. The reviewer’s comment is correct – the submitted version of the draft contains a lot of figures with captions that do not contain a lot of information. I revised thoroughly the captions of most of the figures in the way that readers can understand the figures/diagrams more easily.

“Page 10, the "maximum chat frequency" indicator could be described more clearly : does it represent the activity of the most active user of each chat ?”

Response: Thanks for the suggestion that makes the draft clearer. I edited the sentence in the way that clearly states what “maximum chat frequency” indicates. It appears in line 492 in track change version, line 398 in plain version.

“Page 12, the predominance of the "nazi" topic in International issues raises questions : is the use of nazi-associated words always linked to international politics-discussions, or could it also cover other political (or even non-political) uses as a derogatory term or an insult ?”

Response: Thanks for the questions related to the topic model results. Reviewer is right – it is hard to see a single usage of “Nazi” not co-appearing with any other political keywords as a “political” topic. I revised the draft in the way that corresponds to this decision by revising the figures and related writings.

“Fig 12, it would be interesting to add the Potchamp emote, since its usage is investigated in the previous figure.”

Response: Unfortunately, due to the copyright infringement issue, it was not possible to directly add the screenshot of emotes (as I noted in the response to the editor-suggested revision, I had

to remove the included screenshots from Twitch, including emotes). However, I added the link that authors can use to view how the emotes look like.

Reviewer 2:

"First, the paper claims to provide a "comprehensive" description of political content and actors on Twitch ("This study is the first comprehensive attempt to identify political actors on the platform"), but this claim is almost certainly incorrect. The paper itself acknowledges (in the next paragraph) that it provides "informative snapshots", and the period of observation is both unclear and constrained.

Twitch has been around since 2011, but this analysis appears constrained to 2021 and early 2022. Engagement with or acknowledgement of this limited timeframe is largely absent from the article. Likewise, the paper's label as comprehensive is itself undermined by the important point it makes about incidental exposure, wherein apolitical streamers occasionally leak their political perspectives. Studies of incidental exposure, such as Dreston et al., 2023, show this exposure is formative, so discussion of overtly political actors on a gaming platform is almost certainly incomplete without a deeper engagement of this topic. Certainly, identifying incidental political content on Twitch may not be straightforward (e.g., is something like the "Thanks, Obama" meme political?), but it is a major omission in the descriptive work of this article. Additional care should be taken to resolve this omission and the tension between the paper's claims of comprehensiveness and the role of apolitical streamers in political communication on Twitch."

Response: I want to first thank the reviewer for pointing out this point. The reviewer is absolutely right – the submitted version of this paper is both 1) overstating the contribution and 2) not effectively engaging and acknowledging the weakness and omission. I made a few revisions to address this important issues:

1) *The sentence that was directly quoted by the reviewer is now revised in the way that acknowledges the limitations of the study. It appears in line 711 to 715 in track-change version, line 564 to 568 in plain version.*
2) *The paragraph that includes the sentence that exaggerates contribution of the paper ("Building upon the existing literature, this study aims to comprehensively identify political actors on Twitch and analyze their behavior, particularly focusing on the dynamics within streaming chats") is revised in the way that properly acknowledges the limitations and omissions. It appears in line 172 to 180 in track-change version, line 112 to 118 in plain version.*
3) *The paragraph that discusses the omission of incidental exposure for political content and reasons for the omission is added at the end of section "Why Twitch?". It appears in line 222 to 234 in track-change version, line 158 to 170 in plain version.*

“Second, the datasets and collection process used to support the paper's analysis and conclusions is largely unclear to me. It seems that the political classifier was developed on a very small slice of data, then the content analysis was done on a different dataset collected several months later. And in both cases, the collection window seems quite small. Developing a political classifier from one week of data and then drawing strong conclusions from about 4 months of behavior on a platform that's over ten years old is problematic, especially as this timeframe includes the beginning of the large-scale Russian invasion in Ukraine. As I see it, the core contribution of this work is a description of political content on Twitch; that's a laudable goal, but the constrained and limited detail on data collection impedes this contribution. Perhaps a model paper from a description-focused venue like JQD would be useful, such as Brown et al., 2024 [2].”

Response: I appreciate the reviewer’s observation regarding the constrained timeframe of the data collection and the potential impact on the study’s conclusions. The choice of a one-week window for developing the political classifier and a four-month period for content analysis was guided by the need to capture a manageable yet representative snapshot of political discourse on Twitch. While I recognize that Twitch is an evolving platform and that a longer data collection period could offer additional insights, this study aimed to provide an initial exploration of political content during a period that included significant global events, such as the onset of the Russian invasion of Ukraine.

I acknowledge that the limited timeframe may affect the generalizability of our findings, and I have expanded the conclusion section to clarify this limitation. I have also provided additional details on our data collection process to enhance transparency and address the reviewer’s concern. The following points illustrate revisions to address the issue:

1) *The paragraph at the section “Three questions: Who are they, What types of political content are discussed, and How do they interact” is re-written in the way that properly acknowledges the limitation of the timeframe and data collection window. This revision is largely indebted to the reviewer’s suggestion to go over Brown et al., 2024. I use their articulation of implication as the model for the revision. It appears in line 302 to 315 in track-change version, line 234 to 247 in plain version.*
2) *The subsection “Using Twitch API to retrieve streamer lists” (belong to the section “Question 1: Who are the political streamers?”) is edited in the way that also properly acknowledges the limitation of data collection timeframe and at the same time proposing the rationale for choosing a week timeframe. This revision is also largely indebted to the reviewer’s suggestion to refer Brown et al., 2024 as a model paper, where the justification and acknowledgement of their practical limitations are well illustrated. It appears in line 346 to 351 in track-change version, line 267 to 272 in plain version.*
3) *The paragraph that describes topics related to international politics in the subsection “What is discussed in political Twitch streams?: Topic models of the chat post data” of section “Question 2: What types of political content are discussed during streams?” is revised in the way that addresses the concern of the reviewer’s comment. It appears in line 530 to 540 in track-change version, line 432 to 442 in plain version.*

4) *The paragraphs in the "conclusion" section is revised based on the reviewer's comment. It appears in line 711 to 715, 724 to 727, and 746 to 747 in track-change version, line 564 to 568, 574 to 577, and 591 to 593 in plain version.*

"Third, the content analysis piece of this work is underdeveloped given its centrality to the work's contributions. Multiple resources exist on performing content analysis in social media, with Rodriguez and Storer, 2019 using a topic-modeling based approach similar to this paper [3]. Other resources [4-5] provide guidance on qualitative coding for this kind of content, and Wojcieszak et al., 2022 provides an excellent model for studying political discussion in online spaces. This paper's single-coder approach without a clear codebook guiding the analysis is problematic from a reproducibility standpoint, and it introduces problems around the boundaries of the topics, such as how identity politics differs from US politics. Without a clearer and more rigorous approach to this content characterization, it would be difficult to make claims about the kinds of political content popular on this platform."

Response: I want to first thank the Reviewer for insightful comments regarding the content analysis component of my work. I appreciate your suggestions for improving the clarity and rigor of my approach.

In response to your concerns about the need for a clearer codebook, I have included a comprehensive coding rule for identification and classification of political topics. This coding rule not only defines the boundaries of each category but also emphasizes the importance of rigor in identifying and categorizing political discourse on the platform. The structure of clear coding rules is largely guided by the literature that Reviewer suggested and I want to express gratitude for referring to relevant literature.

The coding rule is included in S4 of Supporting information and the concern of ambiguous boundaries among topic boundaries are well addressed. Again, thanks for the valuable comments that improved the research further.

"Lastly, the statistical analyses of the distributions appears largely qualitative and unclear. For example, the paper claims the skewness associated with the distributions in Figure 5 and elsewhere, but I don't see quantitative evidence of this skew. The descriptions of the distributions also tend to state that the figures are in log scale, but the y axes don't tend to reflect that."

Response: Thanks for raising the point that would make the description of distributions much clearer. The concern of the Reviewer on the description remaining qualitative is very valid. I added the statistical test results that show quantitative evidence of skewness whenever I suggest something related to the skewness of the distributions.

On the log-scale transformation, I refined the explanation of the log-transformation in the figure captions to minimize the misunderstanding – all the figures with log-transformed are only for

x-axis not for y axis. This is to visualize the data in a more meaningful way, as the non-log-transformed version of plots do not convey meaningful information because of the extreme values. For example, the left hand side figure of figure 6 looks like as follows because of the extremely large values from some observations.:

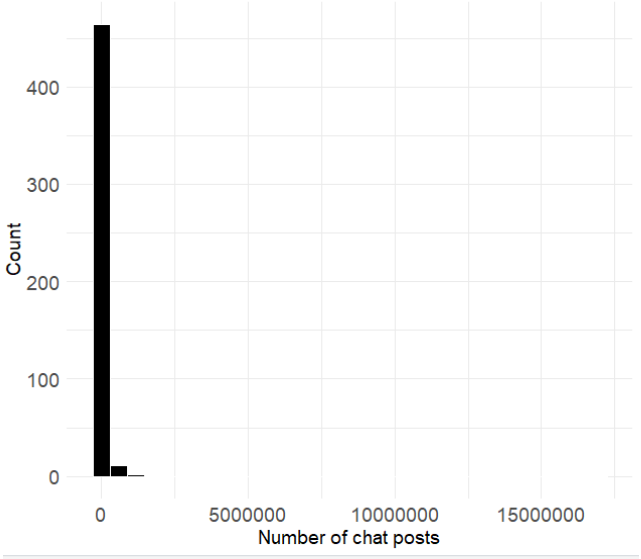

Therefore, I transformed only the x axis to visualize data in the way that we can interpret more meaningful information from the plot as the reported left-hand side figure of figure 6:

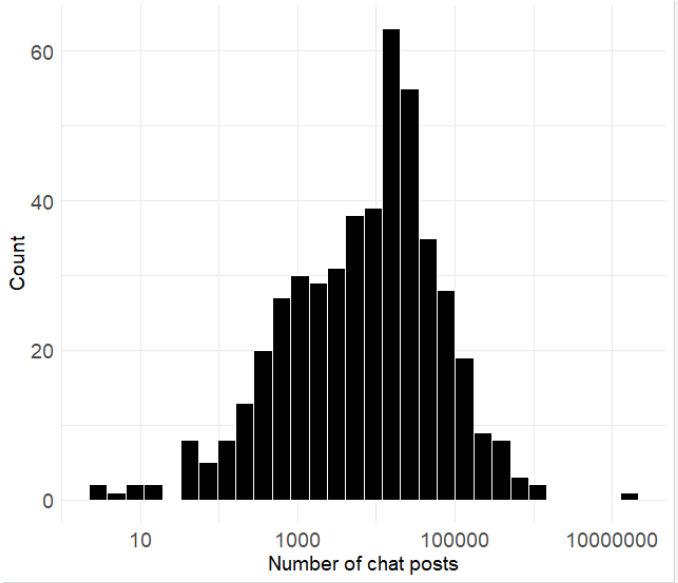

Hope this clarifies the reason I plotted some histograms with only x axis log transformed.

“Generational dynamics have consistently been salient in politics, so the paper's claim that "As generational dynamics have been salient in recent American politics and other countries" seems problematic. Likewise, the paper's characterization that spread of political speech into streaming media and apolitical spaces is new is similarly problematic, as politics from apolitical celebrities or spaces is not new. I think these

points speak to a larger issue about limited engagement with the political science literature."

Response: I deeply appreciate the reviewer for pointing this out. The reviewer's comment is correct – the submitted version of the draft is written in a misleading way without properly relating to the existing literature. To be more specific, I agree that generational dynamics have been consistently important in politics and acknowledge that the phrasing in the original submission may have inadvertently suggested that these dynamics are a recent development.

Also, I agree that political engagement by apolitical celebrities and within entertainment media has a long history and this needs to be related to the existing literature properly. The following points illustrate revisions to address the issue:

The paragraphs of introduction are revised in the way that properly recognize the spread of political content in streaming media and apolitical space is not new. Also citations for existing literature on politics in non-political venues are added. It appears in line 8 to 12 and 27 to 36 in track-change version, line 6 to 10 and 16 to 26 in plain version.

In addition, another paragraph of introduction is revised to properly discuss generational dynamics. Contrary to the submitted version, the paragraph now discusses how generational politics are related to the study without words or sentences that describe the generational politics in a misleading way. It appears in line 68 to 77 in track-change version, line 34 to 43 in plain version.

"I don't understand the network structure in section 7
Why do we have 279 networks for 478 streamers? Do some streamers not have network connections in their chats?"

Response: The paragraph that explains the network structure of the reference network has been written in a way that causes confusion as the Reviewer pointed out. I revised the whole subsection ("Reference network in chat posts within a political stream") in a way that makes the explanation about the network structure clearer and also explains why there are some streamers who do not have a reference network. It appears in line 607 to 631 in track-change version, line 490 and 506 in plain version.

"The ratio of isolates seems low for a platform that is mostly geared toward audience --> streamer engagement"

Response: Thanks for the great insight suggested by the Reviewer. This is absolutely right and I revised the paragraph incorporating this insight to explain the observed pattern. It appears in line 652 to 656 in track-change version, line 512 to 516 in plain version.

"Paragraphs on page 15 seem redundant"

Response: I deeply appreciate pointing out my apparent mistake I made. The first paragraph of page 15 should have been deleted before the submission. I deleted that paragraph. Again, thanks for pointing this out.

“I am unconvinced about the high computational costs of topic modeling. Where does the 5% sampling rate come from? Why not 6% or 2%? Need justification here”

Response: Thank you for raising this important question. The 5% sampling rate was chosen as a pragmatic solution to the computational limitations of processing the entire dataset locally. Given the sheer volume of chat posts, which is over 33 million, analyzing 100% of the data would require resources beyond the available computing infrastructure. The 5% sample was selected based on preliminary test runs to find the manageable size of text data to fit topic models in the local environment. I started from sampling 10% of the number of chat posts and fitting the topic model using R package “stm”, but it was impossible to fit the model due to the high volume of chat post data and the large sized output of topic models. From there, I tested 8%, 7%, and 5% as well. 5% was chosen as the size of chat post data is the most manageable to deal with both text processing and topic modeling. Therefore, although a different sampling rate, such as 2% or 6%, could also be chosen, the 5% rate offered a balance between computational feasibility and preserving the diversity of content for meaningful analysis. I added the explanation to the paper which can be found in 503 to 513 in the track-change version, line 410 to 420 in the plain version.
